# From gaze to proficiency: deep learning-driven prediction of novice performance in laparoscopic training using AOI-dependent metrics

**DOI:** 10.1007/s00464-025-12369-x

**Published:** 2025-12-05

**Authors:** Aseel F. Khanfar, Sanaz Motamedi, Shawn D. Safford, Jason Moore, Jessica Menold, Scarlett Miller

**Affiliations:** 1 Department of Industrial Engineering, The Pennsylvania State University, University Park, PA USA; 2 Department of Industrial Engineering, Yarmouk University, Irbid, Jordan; 3 Division of Pediatric General and Thoracic Surgery, UPMC Children’s Hospital of Pittsburgh, 111 South Front Street, Harrisburg, PA 17101 USA; 4 Department of Mechanical Engineering, The Pennsylvania State University, University Park, PA USA

**Keywords:** Laparoscopic surgery training, Eye-tracking, Visual attention metrics, Computer vision-deep learning, Skill acquisition & random forest prediction

## Abstract

**Background:**

The fundamentals of laparoscopic surgery (FLS) program uses box trainers to develop laparoscopic skills. However, these simulators lack personalized training, real-time objective assessment, and primarily represent adult anatomies, neglecting pediatric cases. To address these limitations, advanced objective evaluations like motion analysis and eye-tracking are needed to track trainees’ progress and provide real-time formative feedback. However, dynamic training environments challenge eye-tracking data extraction due to shifting areas of interest (AOI). This study aimed to extract AOI-dependent and motion metrics for differentiating and predicting trainees’ skill levels across different box trainer anatomies.

**Method:**

Medical students and residents performed the peg transfer task on adult and pediatric box trainers. Computer Vision-Deep Learning (CV-DL) algorithms were integrated with eye-tracking data to automatically detect AOIs and extract AOI-dependent (fixation rates on objects and tools) and motion (tool speed) metrics. K-means clustering was used to differentiate trainees’ skill levels. To predict trainees’ visual behavior, we employed multiple Machine Learning (ML) techniques, including Random Forest, Support Vector Machine, Artificial Neural Networks, and Decision Trees. These methods were used to evaluate which technique could most accurately predict trainees’ visual attention patterns.

**Results:**

The extracted metrics successfully classified novices into High and Mid-Low skill levels, with significant differences in all extracted metrics between visual behavior levels (*p* < 0.05). Random Forest achieved the highest accuracy for visual behavior prediction, highlighting the importance of fixation rates on objects and tool speed as key predictors using Gini importance. Results showed consistency in novices’ visual attention between pediatric and adult box trainers (*p* > 0.05).

**Conclusion:**

The findings from this work are significant, indicating that novices' skill levels may differ even in their early-stage training, and extracted metrics have the potential to classify and predict novices’ skill levels and visual behavior. This is important for customizing and adapting trainees’ training programs to enhance their performance.

**Graphical abstract:**

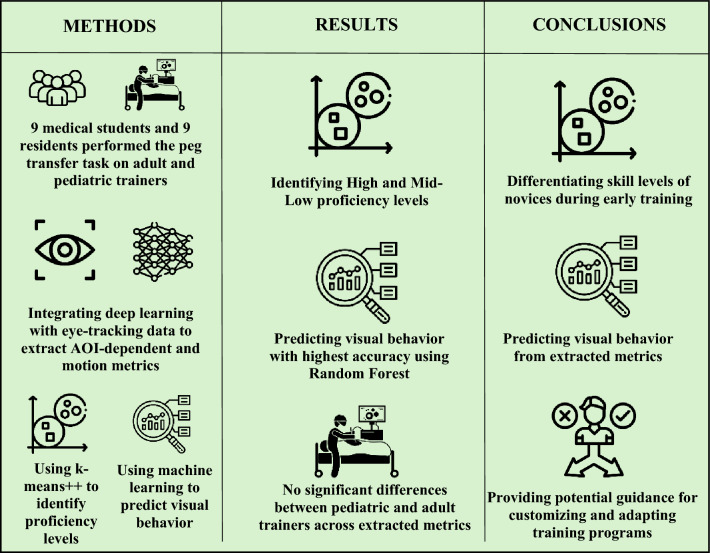

Laparoscopy, a Minimally Invasive Surgery (MIS), is widely used for complex operations, from appendectomies to pancreas resections to adrenalectomies, due to its benefits, including reduced postoperative pain, shorter hospitalization periods, quicker recoveries, and minimal scarring [1–3]. Consequently, laparoscopic cases have increased by 462% from 2000 to 2018, with over 15 million procedures performed annually worldwide [4, 5]. However, the complexity of laparoscopic procedures poses significant challenges for surgeons, such as limited depth perception from two-dimensional monitors and reduced tactile feedback compared to open surgery [6, 7]. Performing the procedure on pediatric patients presents additional challenges due to the small operative space and the increased fragility of the tissue [8]. As such, this procedure requires surgeons to develop advanced skills in perceiving and interpreting visual cues and coordinating their movements to operate effectively [9]. As a result, the traditional “see one, do one, teach one” model is no longer sufficient and has been replaced by Simulation-Based Training (SBT), following the principle of “see one, simulate many, do one completely, and teach everyone.” [10].

SBT has been adopted by laparoscopic surgeons through the Fundamentals of Laparoscopic Surgery (FLS) program to train and evaluate fundamental laparoscopic skills using a series of five tasks on a laparoscopic box trainer (FLS simulator) [11–13]. The peg transfer task, one of the five validated tasks for basic laparoscopic skills [14] is widely used as it is designed to enhance ambidexterity, depth perception, and coordination between both hands, all of which are critical skills in laparoscopic surgery [15, 16]. While the laparoscopic box trainer has demonstrated its effectiveness for training purposes, it has some limitations. One limitation is the lack of a real-time objective assessment [17]. Currently, FLS tasks rely on trained proctors who evaluate performance on-site based on predetermined standards, using speed (time to complete the task) and accuracy (number of errors committed) [11]. Another limitation is that the simulator’s assessment system does not adapt to individual performance, which limits its ability to provide personalized training or feedback [18]. Specifically, variations across novices’ performance in the early stages of training necessitate personalized training programs that more efficiently and effectively contribute to novices’ competency [19, 20]. The third limitation is the scarcity of simulators specifically designed for pediatric patients [21]. The availability of such simulators is important due to the anatomical differences between adult and pediatric patients [8]. Given these limitations, more advanced objective assessment tools should be used to track trainees’ progress and provide constructive feedback to enhance their competency [22–24]. These tools should also be used to predict trainees’ skill levels, enabling personalized training [25, 26]. Furthermore, there is a need for validated pediatric simulators that incorporate such tools for effective training in this specialized field [21]. Among these tools, motion analysis and eye-tracking provide valuable insights into proficiency and visual behaviors to objectively assess trainees’ technical skills [27, 28].

One advanced objective assessment method used in laparoscopic training is motion analysis. This method objectively evaluates trainees' proficiency levels (e.g., novices and experts) by analyzing motion-based metrics of laparoscopic instruments, such as tool speed, path length, and acceleration [29, 30]. These metrics are essential for informing trainees about areas for improvement, allowing them to practice effectively and master the control of laparoscopic instruments [20]. However, motion analysis alone does not capture the cognitive processes involved in surgical training. It lacks the ability to assess how trainees navigate relevant and irrelevant visual information while performing tasks [31]. To address this, eye-tracking technology provides insights into visual attention during surgical procedures [32].

Another tool for advanced objective assessment is eye-tracking, which provides insights into trainees’ visual behavior and cognitive processes, necessary for evaluating their visual attention to relevant targets during training [33]. Eye-tracking is increasingly being applied in medical education as a training tool [34], proficiency assessment [35], and feedback [36]. To derive valuable data from eye-tracking studies, researchers segment the visual field into areas of interest (AOIs) to analyze gaze patterns and make informed decisions [37]. Eye-tracking metrics can be classified into AOI-independent and AOI-dependent eye-tracking metrics [38]. While AOI-independent metrics, such as fixations, saccades, blink rate, and pupil diameter, have been effective in differentiating expertise levels and evaluating mental workload [28, 39], they do not provide direct guidance on how trainees should focus their attention during training [40, 41]. For this reason, in the current work AOI-dependent metrics are used.

AOI-dependent metrics, such as fixation rates and fixation durations within specific AOIs, provide real-time feedback by guiding trainees’ on where to allocate their attention for improving their performance [42]. These metrics reflect both feedback and feedforward gaze behaviors for skill evaluation [43]. Feedback gaze behavior involves tracking relevant targets and tools, with studies showing that experts spent more time gazing at targets, whereas novices allocated more time following the tools [44, 45]. Feedforward gaze behavior is assessed by analyzing eye-tracking metrics from objects or locations where an action is intended before execution. Studies on peg transfer task training found that skilled trainees focus more on future objects than current holding objects, demonstrating greater anticipatory visual control [43, 46]. Despite the effectiveness of these metrics, their application in research remains limited.

Although AOI-dependent metrics could provide formative feedback for trainees, they are rarely used in laparoscopic training [20, 46]. This is mainly due to the challenge of extracting eye-tracking metrics from specific AOIs, as their positions continuously shift based on the gaze data coordination system [47]. Recently, computer vision (CV) algorithms have been explored to automatically extract eye-tracking metrics from dynamic AOIs [20, 38, 48, 49]. For example, a study on peg transfer task training developed a computer vision model to differentiate trainees' skill levels by extracting fixation rates on objects and object-tool combinations [38]. Once extracted, these AOI-dependent metrics, along with motion metrics, can be used in predictive models to classify trainees’ skill levels.

After extracting AOI-dependent and motion metrics and classifying the expertise level of trainees, it is important to predict proficiency based on visual and motion behavior [50]. One approach is the use of machine learning (ML) techniques, which can classify and predict trainees’ proficiency levels (e.g., expert, intermediate, and novice) based on eye-tracking and motion metrics [51]. Previous research applied classifiers such as Support Vector Machine (SVM), Linear Discriminate Analysis (LDA), Artificial Neural Networks (ANNs), Decision Trees (DTs), and Random Forest (RF) to predict expertise levels in laparoscopic training [28, 51]. By determining trainees’ skill levels, these methods can not only monitor their progress but also personalize their training programs to better match their abilities, thus improving their training performance [28, 52].

To sum up, AOI-dependent eye-tracking metrics are effective in differentiating trainees’ skill levels. However, there are significant gaps in the literature including: 1) extracting AOI-dependent eye-tracking metrics in a dynamic environment presents challenges, 2) assessing novices’ proficiency levels during early-stage training using eye-tracking metrics, rather than focusing only on differentiating between experts and novices, and 3) evaluating trainees’ visual attention across different box trainer anatomies (e.g., pediatric and adult box trainers). To address these gaps, a validated computer vision-deep learning (CV-DL) model was employed to extract AOI-dependent eye-tracking, which reflects both feedback and feedforward metrics, along with motion metrics. This study aimed to differentiate skill levels of new trainees, predict their visual behavior during early-stage FLS peg transfer task training, and explore how visual attention may change across different box trainer anatomies. Specifically, the study was designed to answer the following research questions:

***RQ1:* Can AOI-dependent and motion metrics extracted from the CV-DL model classify novice skill proficiency levels?** It was hypothesized that a clustering algorithm would identify different skill levels. It was also hypothesized that fixation rates would be higher on objects for participants at higher proficiency levels and higher on tools for participants at lower proficiency levels. Finally, it was hypothesized that the tool speed would be faster at lower proficiency levels than at higher proficiency levels.

***RQ2:* Can novices’ visual behavior levels be successfully predicted using metrics extracted from the CV-DL model?** It was hypothesized that using machine learning techniques, the visual behavior levels of trainees can be predicted successfully from the extracted metrics.

***RQ3:* Does the type of trainer (pediatric or adult) influence novices’ visual attention across different AOIs during training sessions?** It was hypothesized that the extracted metrics would be higher in the pediatric box trainer than in the adult box trainer, as eye-tracking metrics such as fixations and fixation durations increase with task difficulty [39].

## Materials and methods

### Participants

Nine medical students and nine residents (10 males and 8 females) were recruited for the IRB-approved study (STUDY0023069) at Hershey Medical Center (HMC). The study included residents from general surgery and anesthesiology programs: seven participants were in Postgraduate Year (PGY) 1, and two participants were in PGY3. The PGY3 residents were enrolled in anesthesiology. Two of the participants were left-handed. All participants had completed fewer than two laparoscopic surgeries in their careers and had less than three years of experience. All participants had normal or corrected-to-normal vision.

### Procedure

**Fig. 1:**
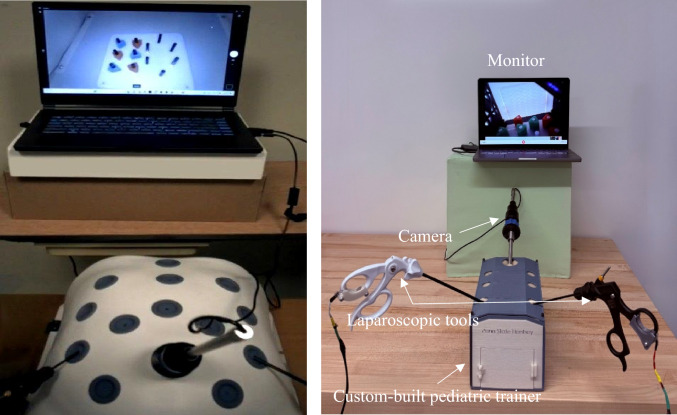
Experimental setup using an adult (left) and pediatric (right) box trainers

At the beginning of the study, randomly selected participants received an explanation of the study objectives and procedures, and informed consent was obtained according to IRB protocol. Afterward, Tobii glasses 3, with a 50Hz sampling rate, were fitted and calibrated for each participant. Participants were then instructed to perform the FLS peg transfer task following FLS standards on two box trainers: one representing adult anatomy with internal dimensions 455 × 395x220 mm, which is a commercially available simulator from Medicinology and Co (see Fig. 1) and another low-fidelity custom-built trainer to simulate pediatric patient anatomy with internal dimensions of 140 × 230 × 126 mm. The custom-built pediatric trainer (see Fig. 1) is a 3D-printable design, following a baseball diamond concept [53] to strategically place ports for optimal triangulation. Maintenance is made easy with the replaceable Brrnoo training suture pad. A camera with a high resolution of 1920 × 1080 was used to ensure visual clarity. To represent the surgical instruments for laparoscopic surgery, 3mm “Laparo Scopy Boxx” pediatric laparoscopic needle holders were used.

Six novices performed the FLS peg transfer task on the adult box trainer, while another six executed the task on the pediatric box trainer. The remaining participants (six novices) completed the task on pediatric and adult box trainers. The order in which participants used the simulators was randomized to avoid order effects. However, statistical analysis confirmed that task order had no significant effect on performance outcomes. Both box trainers were connected to an optical camera that streamed the internal scenes onto a 15-inch monitor. See Fig. 1 for the experimental setup of the pediatric box trainer.

**Fig. 2:**
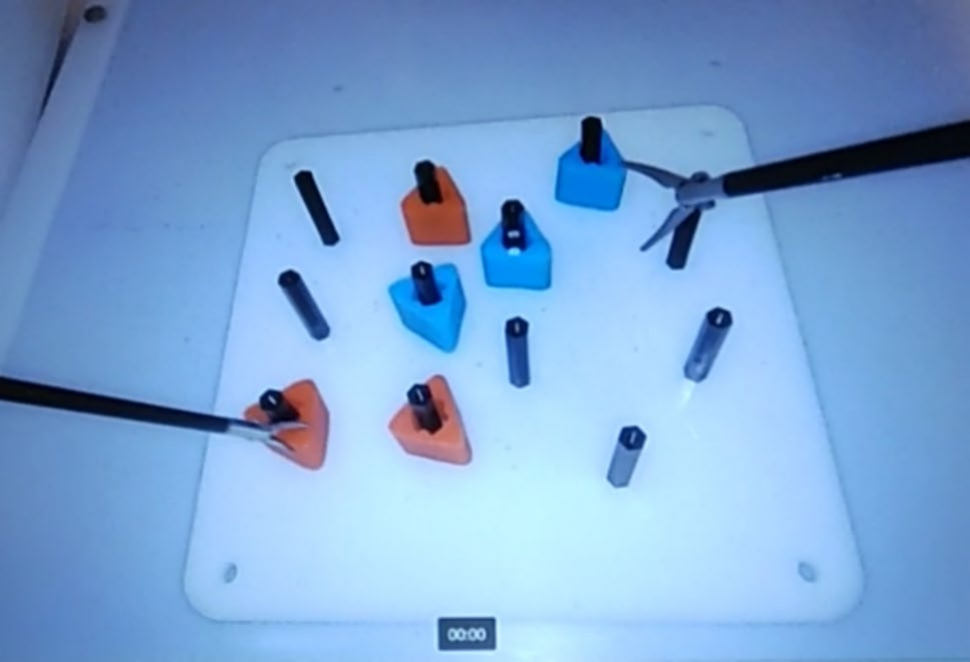
The peg transfer task

**Fig. 3:**
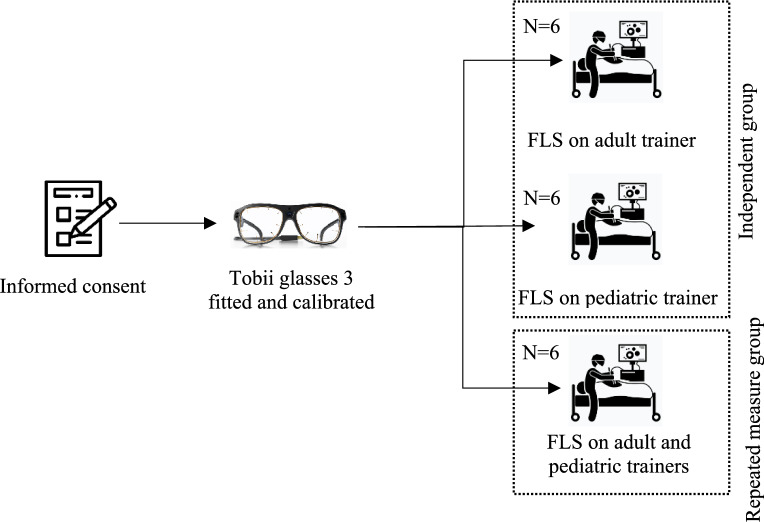
Experimental procedure across pediatric and adult box trainers

Before participants started their task, all six rubber objects should be placed on the same side as the participant’s non-dominant hand. They were then asked to lift each of the six rubber triangles using graspers held by their non-dominant hand and transfer it midair to their dominant hand before placing them on the opposite side of the pegboard (see Fig. 2). Due to time constraints and that participants took an average of 30 min to complete the experiment, participants completed only the first half of the task without reversing, as initially stated by the FLS manual [14]. Upon completing the task on one box trainer, the same procedure was replicated on the other box trainer for the six participants assigned to perform the task on both box trainers. Figure 3 shows the experimental procedure for this work.

### Computer vision- deep learning (CV-DL) model

**Fig. 4:**
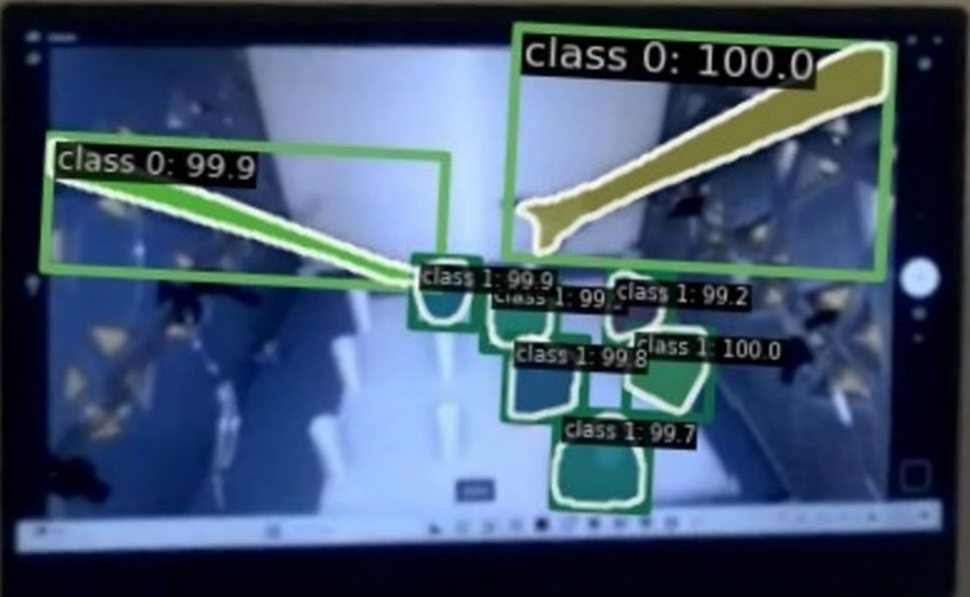
An example of the mask generated using the Mask R-CNN model, extracted from the eye-tracker recording

Mask R-CNN, a CV-DL model, was developed in our previous research [54] to automatically detect AOIs, such as triangles/objects and tools/graspers, during the peg transfer task training, see Fig. 4. It achieved 98.5% precision in classifying AOIs and 74.8% precision in generating masks around the predicted object. To handle potential noise in frame-level object detection, a confidence threshold was applied such that object detections with a probability greater than 0.5 were considered valid. Two test videos (one from an adult simulator and one from a pediatric simulator), including 3889 frames of the peg transfer task training, were used to validate the model [55, 56]. The AOI annotations (e.g., objects and tools) in both videos were generated using the Mask R-CNN model and annotated manually frame-by-frame to serve as ground truth. These annotated frames from each method were then integrated with the eye-tracking data to (1) count the number of times the fixation point fell on the AOIs and (2) assess the level of agreement, frame-by-frame, using Cohen’s kappa (κ) [57] between the model and the Mask R-CNN annotations. The AOI hits were relatively close, and a high level of agreement between the manual annotations and the Mask R-CNN model was achieved, with Cohen’s kappa (κ) exceeding 0.8 for both objects and tools across the pediatric and adult box trainers.

**Fig. 5:**
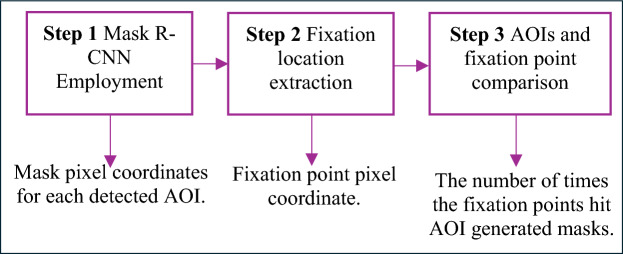
Summary of mask R-CNN employment to extract eye-tracking metrics in this work

Three steps were then applied in this work to extract fixation rates on different areas of interest during the peg transfer task training. Figure 5 shows a summary of the three steps and their outcomes.

In Step 1, Tobii glasses recordings were split into individual frames and then used through a developed Mask R-CNN model to automatically identify and label objects and tools. Generated mask pixel coordinates were extracted for all detected objects in each frame.

In Step 2, fixation point pixel coordinates (*x*, *y*) were obtained from each participant’s video using Tobii Pro Software. To extract fixation points, the Tobii Velocity-Threshold Identification (I-VT) fixation filter [58] was applied at a 70-degree/sec velocity threshold [59, 60], which considers eye-movement below a velocity of 70 degrees/sec as fixation.

Finally, in Step 3, fixation point coordinates were compared to the generated mask coordinates in each frame. If the fixation point fell within five pixels around the contours of triangle objects or tools, the participant was considered to be fixating on the detected AOI. This approach followed Specian Junior, Litchfield [61] recommendation that chosen AOI boundaries can be extended slightly beyond the actual AOI. This extension helps ensure that any fixations near the edges of the AOI are still captured accurately. The number of frames containing fixation points that hit an AOI was divided by the participant’s task completion time to compute fixation rates (measured in frames/second) [38]. Five AOI-dependent eye-tracking metrics, including one representing feedforward behavior, were extracted in addition to one motion metric.

### Metrics

**Fig. 6:**
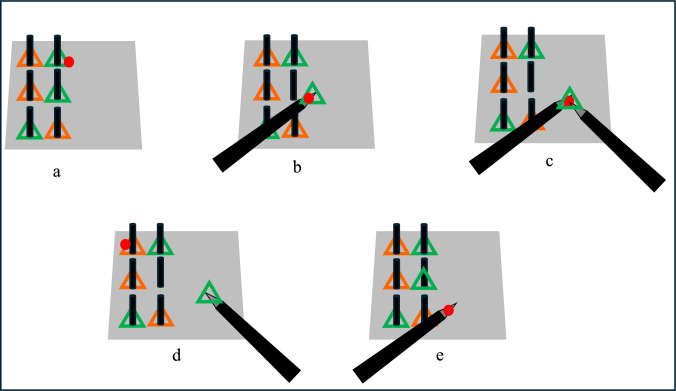
Examples of fixations on different AOIs used in this study where the red circle represents a fixation point: **a** fixation on object, **b** fixation on object being held by one tool, **c** fixation on object being held by two tools, **d** fixation on the next object, **e** fixation on tools

AOI-dependent eye-tracking metrics were extracted based on detected tools and target objects to evaluate novices' visual attention and skill levels during training, e.g., fixation rates on objects and tools, and tool speed. Fixation on objects was further classified into three categories: (1) moving objects manipulated by one tool, (2) moving objects manipulated by two tools, and (3) not moving objects (potentially representing next targets). Hence, in addition to overall fixation rates on objects, fixation rates in these three categories were also computed to provide a more detailed evaluation of novices’ visual behavior. These metrics were derived from previous work [20]. More details about the metrics used in this work are shown below (see Fig. 6):

#### Fixation rates on objects (frames/second)

This feedback metric measures how frequently participants fixate on objects during the task by dividing the number of frames containing fixation points that hit object masks by task completion time.

#### Fixation rates on object-one-tool interaction (frames/second)

This feedback metric measures how frequently participants fixate on moving objects held by one grasper by dividing the number of frames containing fixation points that hit the intersection area between one object and one grasper by task completion time.

#### Fixation rates on object-two-tools interaction (frames/second)

This feedback metric measures how frequently participants fixate on moving objects held by two graspers and may provide insights into the object's transitioning behavior from one tool to another. It is computed by dividing the number of frames containing fixation points that hit the intersection area between one object and two graspers by task completion time.

#### Fixation rates on non-moving objects (frames/second)

This feedforward metric potentially measures participants’ looking-ahead behavior by dividing the number of frames containing fixation points that hit objects outside the intersection area between moving objects and graspers by task completion time.

#### Fixation rates on tools (frames/second)

This feedback metric measures how frequently participants follow the grasper while performing the task by dividing the number of frames containing fixation points that hit graspers in any area outside the intersection area with any objects.

#### Tool speed (pixel/second)

This motion metric measures how quickly the tools are being moved during the task. It is computed by measuring the distance between the tool centroids of two consecutive frames divided by their timestamp difference; this process is repeated for each pair of consecutive frames and then averaged to obtain a single average speed. The final tool speed was obtained by summing the average speed of the left and right graspers.

### Statistical classifier

To predict trainees’ expertise levels from extracted AOI-dependent and motion metrics, four machine learning algorithms were used: (1) Random Forest (RF), an ensemble technique creating multiple decision trees trained on random features and combining these decision trees to predict the class label [62], (2) Support Vector Machine (SVM), which separates different classes of data by finding an optimal hyperplane in the feature space [63], (3) Classification and Regression Trees (CART), recursively dividing features into smaller, non-overlapping regions, with each region associated with a decision tree for classification [64], and (4) Artificial Neural Networks (ANNs), consisting of interconnected nodes arranged in layers, help in understanding the relationships and patterns within the data for prediction purposes [65]. These classifiers were selected due to their wide use on small datasets and their ability to handle nonlinear feature relationships [66, 67]. Multiple classifiers were used to ensure that the best algorithm was identified to predict the expertise levels of trainees from the extracted metrics, as each classifier employs a different approach (e.g., ensemble, margin-based, tree-based, and neural network-based) [68]. Leave-one-out cross-validation (LOOCV) was used to evaluate the performance of machine learning models. LOOCV trains a model on all samples in the dataset, leaving only one sample for testing, and repeats this process where each sample serves as both training and testing points [69]. LOOCV is applied to small datasets to obtain reliable accuracy for prediction models [70].

## Results

This research aims to classify novices’ skill levels and predict their visual behavior using AOI-dependent eye-tracking metrics extracted from the mask R-CNN model and investigate the variations in novices’ visual attention across different box trainer anatomies. The gaze sample percentages for all recordings were at least 87%. Hence, all recordings were included in the analysis. All statistical analysis was conducted using IBM SPSS (V. 29.0) at a significant level of 0.05. A one-way Welch ANOVA was run instead of a one-way ANOVA if the homogeneity of variances was violated. For all analyses in this study, outliers were examined, and analyses were conducted both with and without them. If the results were consistent, the outliers were retained; otherwise, they were excluded. The remainder of this section represents our findings for each of our research questions:

*RQ1:* Can AOI-dependent and motion metrics extracted from the CV-DL model classify novice skill proficiency levels?

To evaluate and differentiate novices’ skill proficiency levels, three steps were employed: clustering participants based on their proficiency levels, interpreting the resulting clusters using task performance, and analyzing differences in visual and motion patterns. These steps are detailed below:

1. Clustering Participants by Proficiency Levels: To classify novices based on their skill levels, we applied an unsupervised ML algorithm, k-means +  + , to behavioral metrics extracted by the CV-DL (Mask R-CNN) model (see Metrics Section). [71]. Based on the elbow method [72] three clusters were determined by plotting the within-cluster sum of squares (WCSS) against the number of clusters, see Fig. 7. Participants were classified as follows: Cluster 1 (*N* = 5), Cluster 2 (*N* = 14), and Cluster 3 (*N* = 5).

**Fig. 7:**
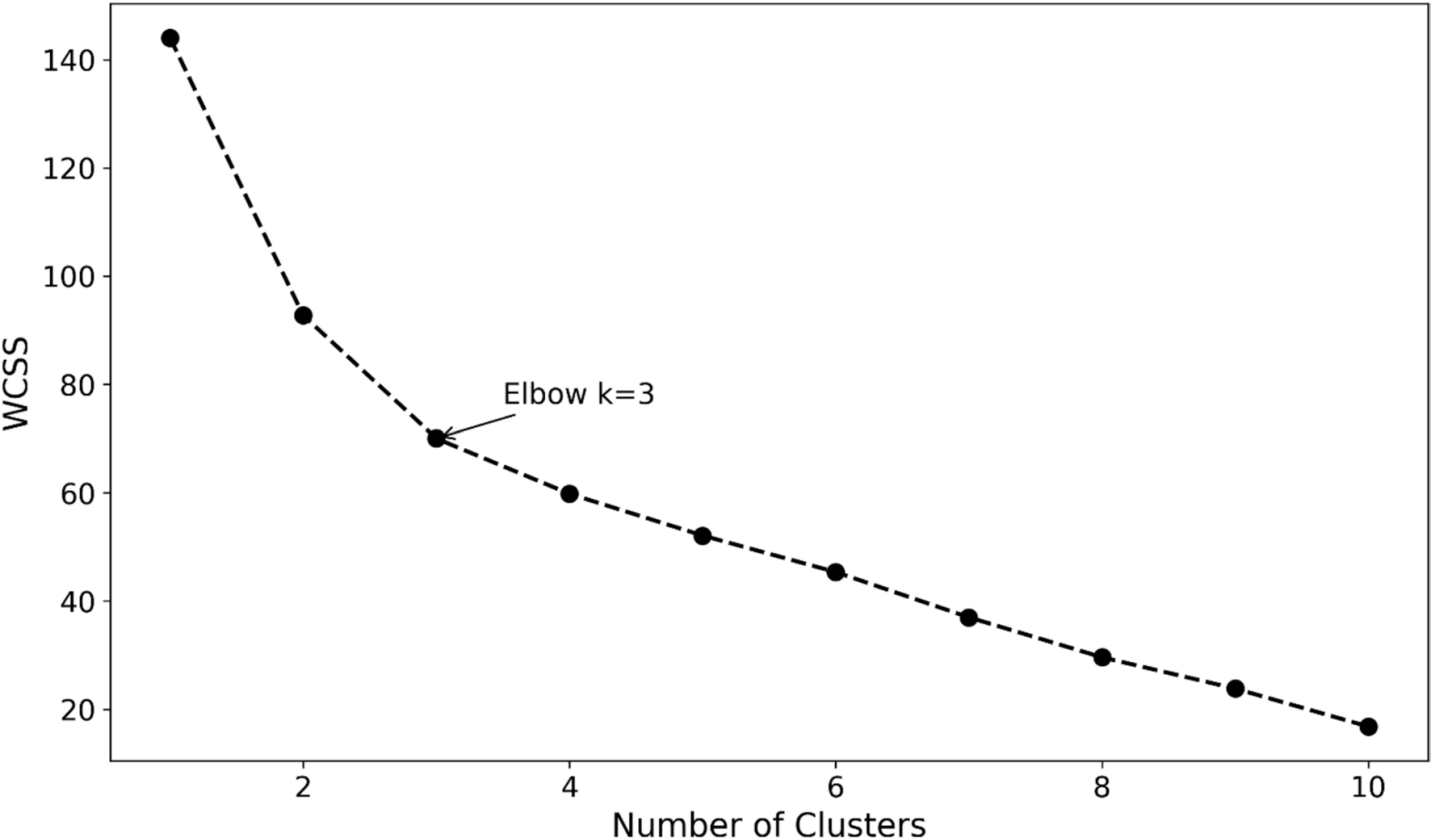
The elbow method of k-means +  + to identify the number of clusters
2. Interpreting Clusters with Task Performance: To interpret skill levels associated with each cluster, we compared task completion times and errors committed across groups. Figure 8 shows the number of errors committed and completion times based on the three defined clusters. A one-way ANOVA was conducted to determine if the errors committed between the three groups were different. The number of errors committed was not statistically significant between the three groups F (2, 21) = 2.893, *p* = 0.563.

**Fig. 8:**
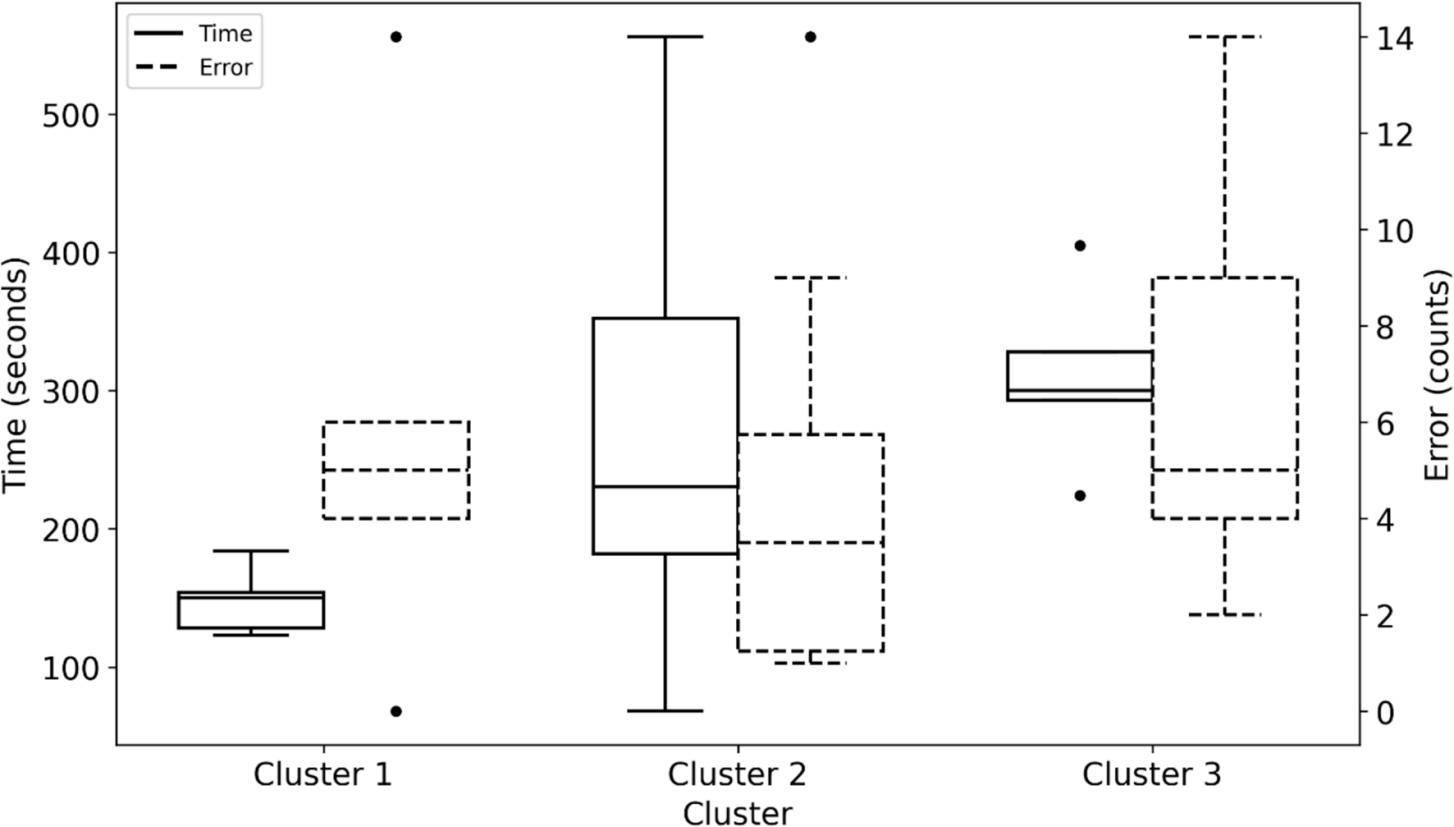
Errors committed and completion times of novices in each identified cluster
  A one-way Welch ANOVA was conducted to determine whether the three clusters' completion times differed. Completion times significantly differed between the three clusters, Welch’s F (2, 9.815) = 14.497, *p* < 0.001. Hames-Howell post-hoc analysis revealed that the mean increase from Cluster 1 to Cluster 2 (− 112.98, 95%CI [− 211.54, -14.42]) was statistically significant, as well as the increase from Cluster 1 to Cluster 3 (− 162.2, 95%CI [− 263.20, − 61.19]). The completion times mean increase from Cluster 2 to Cluster 3 (− 49.21, 95%CI [− 170.52, 72.1]) was not statistically significant, see Table 1. Since there was no statistically significant difference in completion times between Clusters 2 and 3, we expected their proficiency levels to be the same. These results indicate that based on clustering analysis, novices’ visual behavior was classified into three clusters (e.g., Clusters 1, 2, and 3), and these clusters were identified into two skill levels based on the differences in completion times: High (Cluster 1) and Mid-Low (Clusters 2 and 3). Figure 9 shows the radar chart of the AOI-dependent and motion metrics by clusters, visually comparing clusters’ performance across all extracted metrics.

**Table 1:** Pairwise comparisons between the three clusters using the Hames-Howell post-hoc analysis for errors committed and task completion times

Clusters	Cluster 1, Cluster 2		Cluster 1, Cluster 3		Cluster 2, Cluster 3	
Metrics	Difference	*p*-value	Difference	*p*-value	Difference	*p*-value
Number of errors committed	1.300	0.865	− 1.00	0.946	− 2.30	0.618
Completion times	− 112.980	**0.024**	− 162.200	**0.008**	− 49.214	0.556

Significant *p*-values are highlighted in bold

**Fig. 9:**
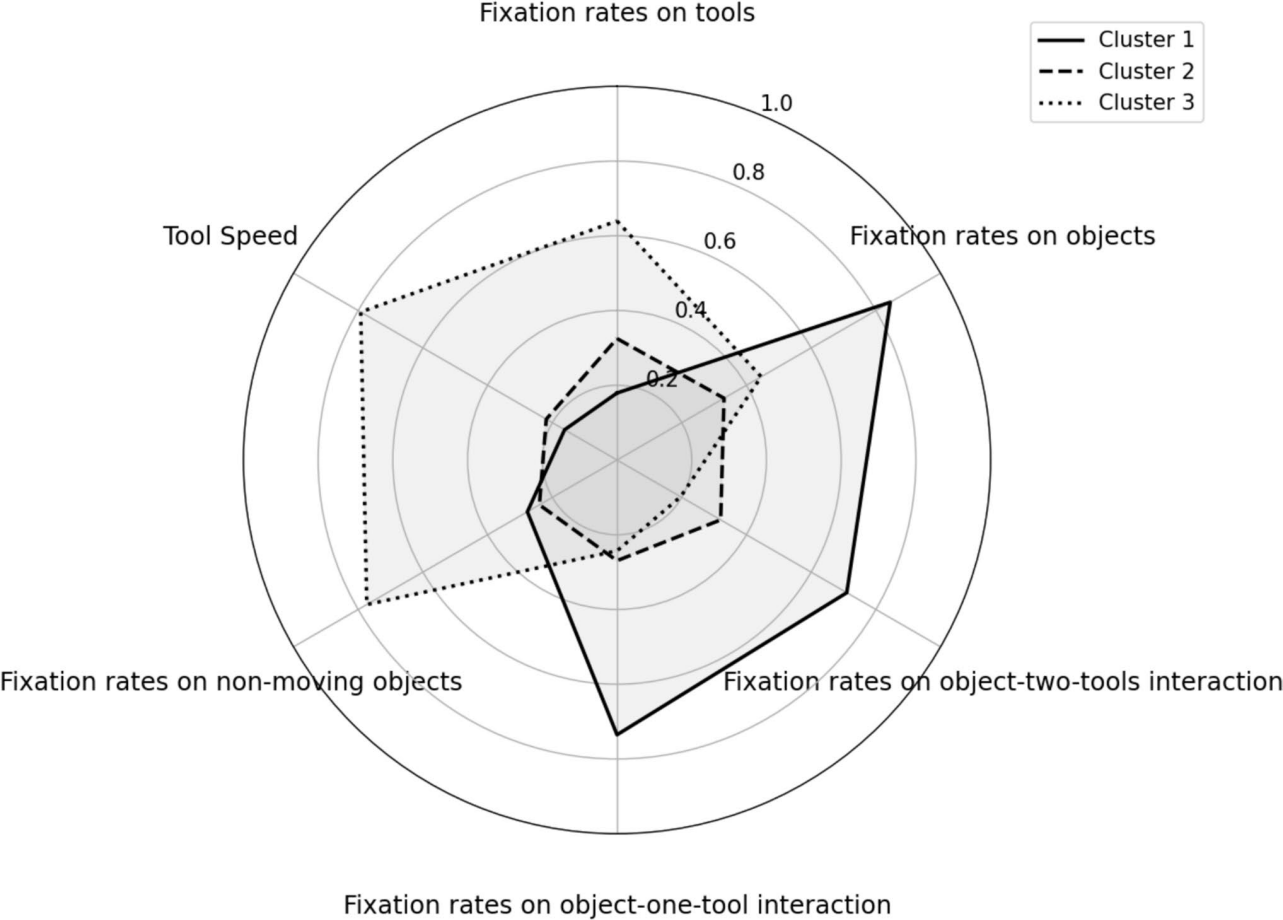
Radar chart of AOI-dependent and motion metrics by clusters
3. Differences in Visual and Movement Metrics: To determine whether the clusters reflected different visual behavior patterns, we conducted six one-way ANOVAs and Tukey Post Hoc Comparisons. There was a statistically significant difference between different visual behavior levels across all extracted metrics (*p* < 0.05), see Fig. 10.

**Fig. 10:**
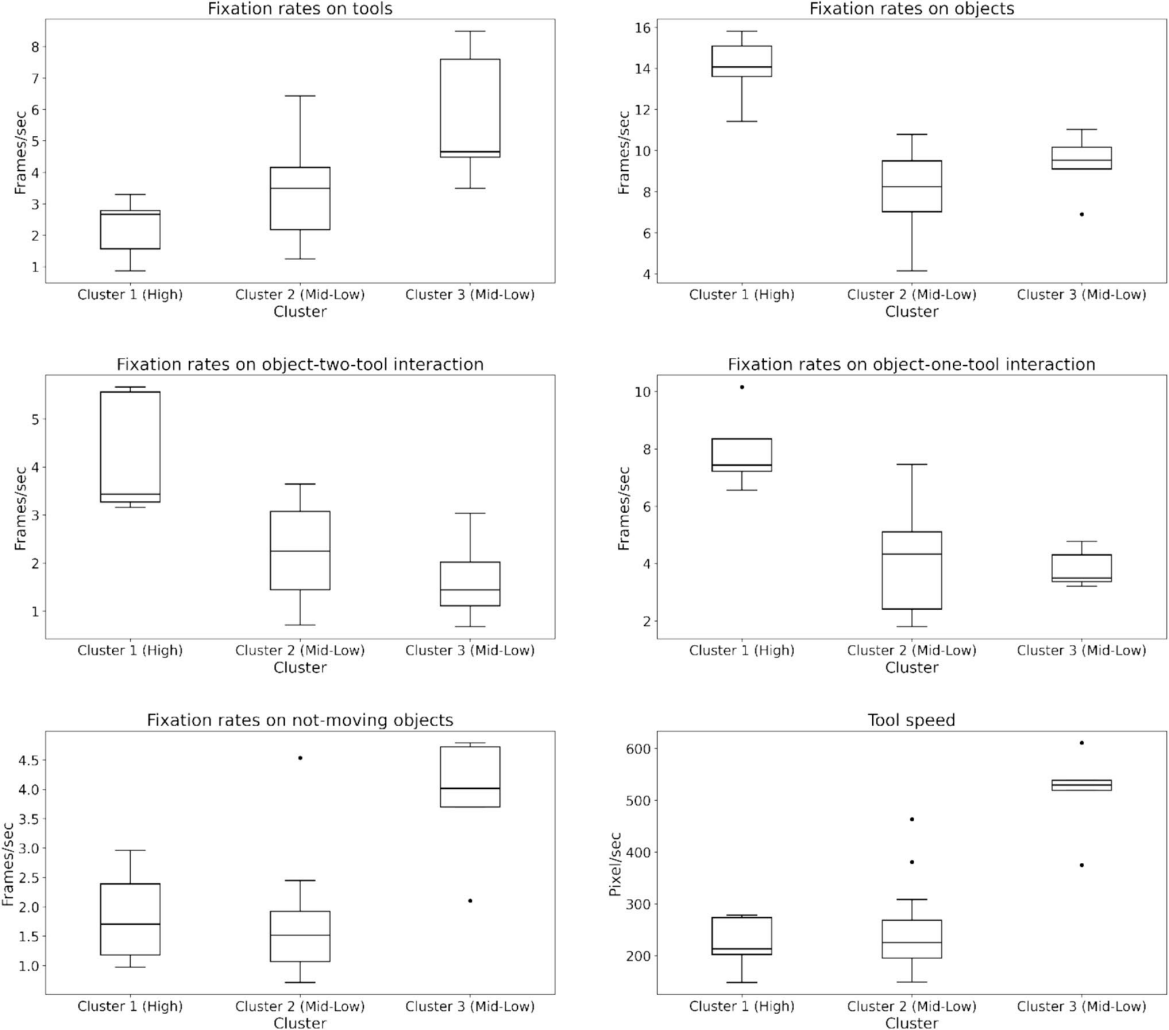
Box plots comparing AOI-dependent metrics and tool speed across the three identified clusters

While one-way ANOVA determined differences between visual behavior levels across all metrics, pairwise comparisons were performed using the Tukey post-hoc test to evaluate the specific differences between each level, as shown in Table 2. Fixation rates on objects, object-one-tool interaction, and object-two-tools interaction significantly differed between Cluster 1 (High)- Cluster 2 (Mid-Low) and Cluster 1 (High)- Cluster 3 (Mid-Low). These metrics significantly increased from Cluster 3 to Cluster 1 and from Cluster 2 to Cluster 1. However, no statistical differences were found between Cluster 2 and Cluster 3. On the other hand, fixation rates on tools, not-moving objects, and tool speed were significantly different between Cluster 1 (High)-Cluster 3 (Mid-Low) and Cluster 2 (Mid-Low)-Cluster 3 (Mid-Low). These metrics significantly increased from Cluster 2 to Cluster 3 and from Cluster 1 to Cluster 3. There was no statistical significance between Cluster 1 and Cluster 2 on tools, not-moving objects, and tool speed metrics.

**Table 2:** Pairwise comparisons between the three skill levels using the Tukey post-hoc test

Clusters	Cluster 1, Cluster 2		Cluster 1, Cluster 3		Cluster 2, Cluster 3	
Metrics	Difference	*p*-value	Difference	*p*-value	Difference	*p*-value
Fixation rate on tools	− 1.10	0.372	− **3.50**	**0.005**	− **2.39**	**0.019**
Fixation rate on objects	**5.98**	**< 0.001**	**4.64**	**0.001**	− 1.33	0.34
Fixation rate on object-one tool interaction	**3.89**	**< 0.001**	**4.11**	**< 0.001**	0.21	0.957
Fixation rate on object-two tools interaction	**1.94**	**0.004**	**2.56**	**0.002**	0.61	0.485
Fixation rate on not moving objects	0.15	0.951	**-2.02**	**0.009**	− **2.17**	**< 0.001**
Tool speed	− 26.18	0.812	− **291.55**	**< 0.001**	− **265.37**	**< 0.001**

Significant *p*-values are highlighted in bold

While these results support our hypothesis that fixation rates on objects would be higher for more-skilled novices and fixation rates on tools and tool speed would be higher for less-skilled ones, the results for the fixation rates on not-moving objects refute our hypothesis. Also, the results show that even clusters with the same skill levels may show different visual behavior and movement skill levels.

*RQ2*: Can novices’ visual behavior levels be successfully predicted using metrics extracted from the CV-DL model?

Four ML algorithms were used to predict novices’ visual behavior levels from extracted metrics: (1) Random Forest (RF), (2) Support Vector Machine (SVM), (3) Classification and Regression Trees (CART), and (4) Artificial Neural Networks (ANNs). LOOCV was employed, where each sample in the dataset was used once as a test set while the remaining samples were used for training. This process was repeated until all samples had served as training and testing points. Accuracy and F1-score were used to evaluate the algorithm's performance. Accuracy presents the proportion of correctly classified predictions out of the total data, while the F1-score is the harmonic mean of precision and recall [73]. The results showed that RF achieved the highest accuracy and F1-score (83.33%, 0.8333, respectively) in predicting novices’ visual behavior levels, followed by ANNs (79.16%, 0.7647), SVM (75%, 0.6554), and CART (70.83%, 0.5132), with the least accuracy and F1-score.

**Fig. 11:**
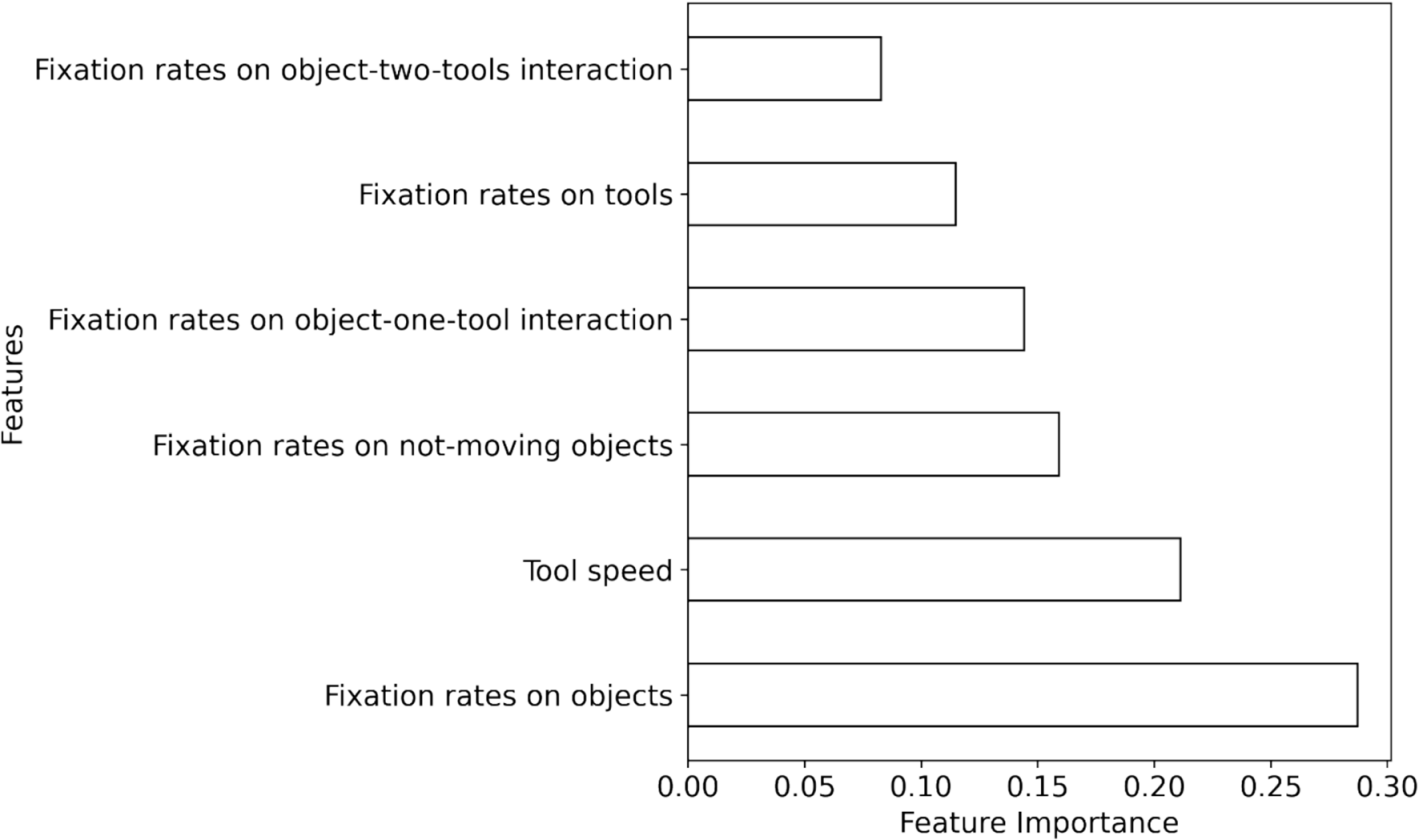
The importance of metrics in predicting visual behavior levels in the RF model based on Gini importance

For evaluating the significance of each predictor (AOI-dependent metrics and tool speed) in the RF model, Gini (impurity) importance was used as a feature selection method [74]. Gini importance measures how much a specific metric contributes to decreasing impurity/ uncertainty when used for splits in RF decision trees [74]. As shown in Fig. 11, fixation rates on objects and tool speed were the most influential features for predicting visual behavior levels, followed by fixation rates on not-moving objects and fixation rates on object-one-tool interaction. Fixation rates on tools and object-two-tools interaction were the least important features. These results support our hypothesis that extracted metrics that reflected feedback and motion metrics would successfully predict novices’ visual behavior levels.

*RQ3:* Does the type of trainer (pediatric or adult) influence novices’ visual attention across different AOIs during training sessions?

Using a paired-sample *t*-test for the repeated measures group, all metrics showed no statistically significant difference between pediatric and adult box trainers (*p* > 0.05). For the independent measures group, an independent sample t-test was run. The independent group found the same results, with no statistical difference between pediatric and adult box trainers across all metrics (*p* > 0.05). These results refute our hypothesis that eye-tracking and motion metrics would be higher in pediatric trainer than in adult trainer.

## Discussion

The goal of this research was to classify novices’ skill levels, predict their visual behavior, and explore variations in novices’ visual attention across different types of box trainers using AOI-dependent and motion metrics from the Mask R-CNN model. The findings from the first research question support our hypothesis that AOI-dependent and motion metrics would successfully classify novices’ skills into multiple levels. Three clusters were identified through clustering analysis, representing different visual behavior levels. Similar results were found in [20], where three clusters were also identified, corresponding to three skill levels. Differences in completion times among these clusters indicated two distinct skill levels: High (Cluster 1) and Mid-Low (Clusters 2 and 3). ANOVAs revealed significant differences in visual behavior between the three clusters across all AOI-dependent and motion metrics. For Cluster 1 (High), fixation rates on objects, fixation rates on object-one-tool interaction, and fixation rates on object-two-tools interaction showed a consistent pattern, where participants at Cluster 1 (High) had significantly higher values than Levels 2 and 3 (Mid-Low). In addition, fixation rates on tools and tool speed showed the opposite pattern, Cluster 1 (High) had significantly lower values than Cluster 3 (Mid-Low) across these metrics. These results indicate that skilled novices can exhibit similar visual behavior to experts, as previous research indicated that experts allocated their attention more to relevant objects during laparoscopic training [44, 45], and novices tend to fixate more on tracking tools, showing “tool-following behavior” and faster tool speed [30]. In general, fixation rates on object-one-tool interaction were higher than fixation rates on object-two-tools interaction, indicating that fixating on the object while two tools hold it occurs less frequently, such as transitioning the object from one tool to another, with participants concentrating more during initial grasping and manipulation.

On the other hand, fixation rates on not-moving objects were also significantly lower for participants at Cluster 1 (High) compared to those at Cluster 3 (Mid-Low). This result contradicts previous research indicating that more-skilled participants tend to look ahead more frequently than less-skilled ones [20, 43]. It’s worth noting that error incidents were included in this research, and participants at Cluster 3 (Mid-Low) committed more errors on average than those at Cluster 1 (High), although this difference was not statistically significant. This might lead participants to look more at the objects that have been dropped and are not being held by any tools. Results showed that fixation rates on not-moving objects for all skill levels are approximately low. This is reasonable due to their initial trials on the peg transfer task, and perhaps more practice is needed to develop the ability to anticipate future actions as experts.

Interestingly, while Clusters 2 and 3 showed the same skill level based on performance measures, they demonstrated significant differences in their visual behavior and movement skills in terms of fixation rates on tools and tool speed. The results showed that Cluster 2 had significantly lower values across these metrics than Cluster 3. This suggests that participants in Cluster 2 may fall somewhere between low and high skill levels. More studies are necessary to investigate the performance of participants at intermediate skill levels [38].

Results from the second research question are consistent with our hypothesis that visual behavior can be successfully predicted using metrics derived from the Mask R-CNN model. Our findings demonstrated that among the four ML models executed, Random Forest was the one that predicted visual behavior with approximately a good accuracy and an F1-score of 83.33%. Random Forest is considered one of the top classifiers and has been shown to perform well compared to other models, such as Support Vector Machines and Neural Networks, to name a few [75]. This may be attributed to the fact that RF builds multiple decision trees and combines their outputs to improve prediction performance [76].

Besides tool speed, results also showed that fixation rates on objects, fixation rates on non-moving objects, and fixation rates on object-one-tool interaction were the most significant predictors of visual behavior. These results provide evidence that these metrics can be used in early-stage training to predict visual behavior, and feedforward, feedback, and motion metrics are all important for this prediction. Our results slightly differed from those of Kulkarni, Deng [20] who found feedforward metrics to be the most important features for predicting skill levels. However, they predicted skill levels that reflect skill acquisition progression, while our work predicted the differences in novices' visual behavior in their first trial training. Identifying the most important features for predicting novices’ visual behavior is important to enhance skill assessment in medical training, potentially provide real-time formative assessment, and support the development of personalized training to improve competency. Integrating AOI-dependent and motion metrics can support formative assessment by tracking where trainees focus their attention during training and how they perform tasks during training, rather than evaluating them only after the session. This enables identifying areas where trainees struggle and provides real-time guidance to support skill development.

The results of the final research question showed no differences between pediatric and adult box trainers. This result does not align with our hypothesis that metrics would be higher in a pediatric box trainer than in an adult box trainer, as previous research indicates that a pediatric box trainer was more challenging than an adult trainer [50]. Consequently, eye-tracking metrics were expected to increase with more complex tasks [39]. Our study suggests that the task demands between both trainers are the same for novices. One potential explanation could be that the custom-built pediatric trainer might not accurately simulate pediatric anatomy and the associated task difficulty. Additionally, the small sample size may limit the ability to detect statistically significant differences between the two box trainers.

## Limitations and future work

While this study shows promising results in classifying and predicting novices’ skill levels, this work has several limitations. While the Mask R-CNN model showed a very good agreement with manual frame-by-frame annotation, it generates mask contours around the detected objects with 74.8% precision. This indicates the existence of potential failure in detecting objects. However, this precision may remain adequate for training feedback. Since eye-tracking captures at high frame rates (e.g., 25 frames/second), AOI predictions are continuously updated, and a large number of correctly detected AOIs accumulate over time. This helps ensure that the feedback remains meaningful for trainees. Additionally, the model’s development was specific to the peg transfer task, and the findings cannot be generalized to other FLS tasks. Another limitation is that data collection was conducted at one large academic institution, which limited the sample size for recruitment. This study did not include expert participants, such as FLS-certified residents, to allow for direct comparison with novices. Furthermore, participants only completed the first half of the peg transfer task due to time constraints, with one trial only on each simulator. To address these limitations, future work may consider further developing the CV-DL model to achieve higher precision, potentially providing a more precise metric estimation. Further studies should focus on developing models and exploring relevant metrics for other FLS tasks, such as suturing. Moreover, large-scale studies involving experts and novices from multiple institutions are necessary to validate the study findings. While this work focused on investigating the differences in novices’ skill levels during early-stage training, further research may use eye-tracking metrics extracted by CV-DL models to evaluate how these metrics change as trainees progress in skill acquisition. Finally, fixation rates on not-moving objects were intended to assess novices’ looking-ahead behavior. Given that the number of errors committed by all skill levels was approximately the same, this facilitated the interpretation of this metric because dropping objects during training shifted trainees’ visual behavior to that object, potentially leading to an inaccurate metric estimation. Further research is important to find a way to extract fixation rates on dropping objects to enhance the skill evaluation.

## Conclusions

This research aimed to categorize novices’ skill levels, predict their visual behavior, and evaluate the variations in visual attention across pediatric and box trainers during peg transfer task training using AOI-dependent and motion metrics. The first main finding was that eye-tracking and motion metrics were successfully used to classify novices’ skills into two levels using the clustering algorithm, and there were significant differences in visual behavior levels across all metrics. Second, Random Forest accurately predicted visual behavior levels, highlighting the importance of fixation rates on objects and tool speed as key predictors. Finally, visual attention for novices remained the same across pediatric and adult box trainers. We can conclude that novices present different skill levels and visual behavior even in the early training stages. Metrics extracted from the Mask R-CNN model have great potential to be integrated into training sessions to provide novices with real-time feedback on where to look (e.g., looking to relevant objects during training) and how to allocate their visual attention (e.g., fixating more on objects than tools). ML models can be further employed to predict novices' skill levels and visual behavior with appropriate features, which help customize and adapt their training program. Further studies should validate the effectiveness of custom-built pediatric box trainers in simulating pediatric patient anatomies.

## References

[1] Chan KS, Wang ZK, Syn N, Goh BK (2021) Learning curve of laparoscopic and robotic pancreas resections: a systematic review. Surgery 170:194–206 33541746 10.1016/j.surg.2020.11.046

[2] Ranucci CM, Lai Q, Quaresima S, Paganini AM, Celani S, Rossi M, Tebala GD, Di Saverio S (2023) New trends in laparoscopic procedures in the emergency abdominal surgery. Springer, The high-risk surgical patient, pp 269–278

[3] Patil Jr M, Gharde P, Reddy K, Nayak K, Patil M (2024) Comparative analysis of laparoscopic versus open procedures in specific general surgical interventions. Cureus 16 10.7759/cureus.54433 PMC10951803 38510915

[4] Blencowe NS, Waldon R, Vipond MN (2018) Management of patients after laparoscopic procedures. BMJ 360 10.1136/bmj.k120 29437677

[5] Bingmer K, Ofshteyn A, Stein SL, Marks JM, Steinhagen E (2020) Decline of open surgical experience for general surgery residents. Surg Endosc 34:967–972 31183795 10.1007/s00464-019-06881-0

[6] Schmidt MW, Fan C, Köppinger KF, Schmidt LP, Brechter A, Limen EF, Vey JA, Metz M, Müller-Stich BP, Nickel F (2024) Laparoscopic but not open surgical skills can be transferred to robot-assisted surgery: a systematic review and meta-analysis. World J Surg 48:14–28 38686793 10.1002/wjs.12008

[7] Schostek S, Schurr MO, Buess GF (2009) Review on aspects of artificial tactile feedback in laparoscopic surgery. Med Eng Phys 31:887–898 19595620 10.1016/j.medengphy.2009.06.003

[8] Yamada K, Muto M, Murakami M, Onishi S, Sugita K, Yano K, Harumatsu T, Nishida N, Nagano A, Kawano M (2023) An analysis of the correlation between the efficacy of training using a high-fidelity disease-specific simulator and the clinical outcomes of laparoscopic surgery for congenital biliary dilatation in pediatric patients. Int J Comput Assist Radiol Surg 18:55–61 36374397 10.1007/s11548-022-02793-y

[9] DeLucia PR, Betts ET (2008) Separated versus integrated displays in minimally-invasive surgery. Proceedings of the human factors and ergonomics society annual meeting, SAGE publications, Los Angeles, CA, pp 894–897

[10] Vozenilek J, Huff JS, Reznek M, Gordon JA (2004) See one, do one, teach one: advanced technology in medical education. Acad Emerg Med 11:1149–1154 15528578 10.1197/j.aem.2004.08.003

[11] Okrainec A, Soper NJ, Swanstrom LL, Fried GM (2011) Trends and results of the first 5 years of fundamentals of laparoscopic surgery (FLS) certification testing. Surg Endosc 25:1192–1198 20872021 10.1007/s00464-010-1343-0

[12] Soper NJ, Fried GM (2008) The fundamentals of laparoscopic surgery: its time has come. Bull Am Coll Surg 93:30–32 19469354

[13] Peters JH, Fried GM, Swanstrom LL, Soper NJ, Sillin LF, Schirmer B, Hoffman K, Committee SF (2004) Development and validation of a comprehensive program of education and assessment of the basic fundamentals of laparoscopic surgery. Surgery 135:21–27 14694297 10.1016/s0039-6060(03)00156-9

[14] FLS (2014) FLS manual skills written instructions and performance guidelines

[15] Fried GM, Feldman LS, Vassiliou MC, Fraser SA, Stanbridge D, Ghitulescu G, Andrew CG (2004) Proving the value of simulation in laparoscopic surgery. Ann Surg 240:518–528 15319723 10.1097/01.sla.0000136941.46529.56 PMC1356442

[16] Derossis AM, Fried GM, Sigman HH, Barkun JS, Meakins JL (1998) Development of a model for training and evaluation of laparoscopic skills. Am J Surg 175:482–487 9645777 10.1016/s0002-9610(98)00080-4

[17] Aggarwal R, Moorthy K, Darzi A (2004) Laparoscopic skills training and assessment. J British Surg 91:1549–1558 10.1002/bjs.4816 15547882

[18] Perrenot C, Perez M, Tran N, Jehl J-P, Felblinger J, Bresler L, Hubert J (2012) The virtual reality simulator dV-trainer® is a valid assessment tool for robotic surgical skills. Surg Endosc 26:2587–2593 22476836 10.1007/s00464-012-2237-0

[19] Sachdeva AK, Loiacono LA, Amiel GE, Blair PG, Friedman M, Roslyn JJ (1995) Variability in the clinical skills of residents entering training programs in surgery. Surgery 118:300–309 7638747 10.1016/s0039-6060(05)80338-1

[20] Kulkarni CS, Deng S, Wang T, Hartman-Kenzler J, Barnes LE, Parker SH, Safford SD, Lau N (2023) Scene-dependent, feedforward eye gaze metrics can differentiate technical skill levels of trainees in laparoscopic surgery. Surg Endosc 37:1569–1580 36123548 10.1007/s00464-022-09582-3 PMC11062149

[21] Joosten M, de Blaauw I, Botden SM (2022) Validated simulation models in pediatric surgery: a review. J Pediatr Surg 57:876–886 35871858 10.1016/j.jpedsurg.2022.06.015

[22] Sim JH (2017) Focusing on formative assessments: a step in the right direction. Acad Med 92(3):275 10.1097/ACM.0000000000001547 28221225

[23] Oropesa I, Sánchez-González P, Lamata P, Chmarra MK, Pagador JB, Sánchez-Margallo JA, Sánchez-Margallo FM, Gómez EJ (2011) Methods and tools for objective assessment of psychomotor skills in laparoscopic surgery. J Surg Res 171:e81–e95 21924741 10.1016/j.jss.2011.06.034

[24] Ng IK, Mok SF, Teo D (2024) Competency in medical training: current concepts, assessment modalities, and practical challenges. Postgraduate Med J qgae023 10.1093/postmj/qgae023 38376156

[25] Menekse Dalveren GG, Cagiltay NE (2020) Distinguishing intermediate and novice surgeons by eye movements. Front Psychol 11:542752 33013592 10.3389/fpsyg.2020.542752 PMC7511664

[26] Ahmed A, Abid MA, Bhatti NI (2016) Balancing standardized testing with personalized training in surgery. Adv Med Educ Pract. 10.2147/AMEP.S122312 28096706 10.2147/AMEP.S122312 PMC5207203

[27] Van Hove P, Tuijthof G, Verdaasdonk E, Stassen L, Dankelman J (2010) Objective assessment of technical surgical skills. Br J Surg 97:972–987 20632260 10.1002/bjs.7115

[28] Richstone L, Schwartz MJ, Seideman C, Cadeddu J, Marshall S, Kavoussi LR (2010) Eye metrics as an objective assessment of surgical skill. Ann Surg 252:177–182 20562602 10.1097/SLA.0b013e3181e464fb

[29] Sánchez-Margallo JA, Sánchez-Margallo FM, Oropesa I, Enciso S, Gómez EJ (2017) Objective assessment based on motion-related metrics and technical performance in laparoscopic suturing. Int J Comput Assist Radiol Surg 12:307–314 27423649 10.1007/s11548-016-1459-3

[30] Stefanidis D, Scott DJ, Korndorffer JJR (2009) Do metrics matter? Time versus motion tracking for performance assessment of proficiency-based laparoscopic skills training. Simul Healthc 4:104–108 19444048 10.1097/SIH.0b013e31819171ec

[31] Wolfe JM (2021) Guided search 6.0: an updated model of visual search. Psychon Bull Rev 28:1060–1092 33547630 10.3758/s13423-020-01859-9 PMC8965574

[32] Liu K, Luo S, Wang X, Cao J, Guo Y, Zhang Y, Li B, Zhang L, Wang X (2024) Objective assessment of visual attention in orthognathic surgery training based on eye tracking. J Cranio Maxillofac Surg 52:65–70 10.1016/j.jcms.2023.08.017 37884435

[33] Franchak JM (2020) Visual exploratory behavior and its development. Elsevier, Psychology of learning and motivation, pp 59–94

[34] Wilson MR, Vine SJ, Bright E, Masters RS, Defriend D, McGrath JS (2011) Gaze training enhances laparoscopic technical skill acquisition and multi-tasking performance: a randomized, controlled study. Surg Endosc 25:3731–3739 21671125 10.1007/s00464-011-1802-2 PMC3213335

[35] Khanfar AF, Motamedi S, Safford SD, Moore J, Menold J, Miller S (2025) Visual attention and cognitive workload using different laparoscopic box trainers and mixed-reality feedback. Surg Endosc. 10.1007/s00464-025-11881-4 40624424 10.1007/s00464-025-11881-4 PMC12287135

[36] Ahmidi N, Hager GD, Ishii L, Fichtinger G, Gallia GL, Ishii M (2010) Surgical task and skill classification from eye tracking and tool motion in minimally invasive surgery. medical image computing and computer-assisted intervention–MICCAI 2010: 13th International conference, Beijing, China, September 20–24, 2010, Proceedings, Part III 13, Springer, pp 295–302 10.1007/978-3-642-15711-0_37 20879412

[37] Cummins RG (2017) Eye tracking. In: Matthes J et al (eds) The international encyclopedia of communication research methods. John Wiley & Sons. Inc.

[38] Deng S, Kulkarni C, Wang T, Hartman-Kenzler J, Barnes LE, Henrickson Parker S, Safford SD, Rajamohan S, Lau NK (2021) Differentiating laparoscopic skills of trainees with computer vision based metrics. Proceedings of the human factors and ergonomics society annual meeting, SAGE Publications, Los Angeles, CA, pp 304–308

[39] Dalveren GGM, Cagiltay NE (2018) Using eye-movement events to determine the mental workload of surgical residents. J Eye Movement Res 11 10.16910/jemr.11.4.3 PMC7903203 33828705

[40] Khan RS, Tien G, Atkins MS, Zheng B, Panton ON, Meneghetti AT (2012) Analysis of eye gaze: do novice surgeons look at the same location as expert surgeons during a laparoscopic operation? Surg Endosc 26:3536–3540 22733194 10.1007/s00464-012-2400-7

[41] Deng S, Oh J, Wang T, Parker SH, Lau NK (2023) Assessing laparoscopic surgical skills of trainees with scene independent and dependent eye gaze metrics. Proceedings of the human factors and ergonomics society annual meeting, SAGE Publications Sage CA: Los Angeles, CA, pp 21695067231192642

[42] Leff DR, James DR, Orihuela-Espina F, Kwok K-W, Sun LW, Mylonas G, Athanasiou T, Darzi AW, Yang G-Z (2015) The impact of expert visual guidance on trainee visual search strategy, visual attention and motor skills. Front Hum Neurosci 9:526 26528160 10.3389/fnhum.2015.00526 PMC4604246

[43] Liu S, Donaldson R, Subramaniam A, Palmer H, Champion CD, Cox ML, Appelbaum LG (2021) Developing expert gaze pattern in laparoscopic surgery requires more than behavioral training. J Eye Movement Res 14 10.16910/jemr.14.2.2 PMC8019143 33828818

[44] Wilson M, McGrath J, Vine S, Brewer J, Defriend D, Masters R (2010) Psychomotor control in a virtual laparoscopic surgery training environment: gaze control parameters differentiate novices from experts. Surg Endosc 24:2458–2464 20333405 10.1007/s00464-010-0986-1 PMC2945464

[45] Law B, Atkins MS, Kirkpatrick AE, Lomax AJ (2004) Eye gaze patterns differentiate novice and experts in a virtual laparoscopic surgery training environment. Proceedings of the 2004 symposium on eye tracking research & applications, pp 41–48

[46] Kulkarni CS (2021) Context dependent gaze metrics for evaluation of laparoscopic surgery manual skills. Virginia Tech

[47] Vansteenkiste P, Cardon G, Philippaerts R, Lenoir M (2015) Measuring dwell time percentage from head-mounted eye-tracking data–comparison of a frame-by-frame and a fixation-by-fixation analysis. Ergonomics 58:712–721 25529829 10.1080/00140139.2014.990524

[48] Garcia-Garcia A, Orts-Escolano S, Oprea S, Villena-Martinez V, Martinez-Gonzalez P, Garcia-Rodriguez J (2018) A survey on deep learning techniques for image and video semantic segmentation. Appl Soft Comput 70:41–65

[49] Pouyanfar S, Sadiq S, Yan Y, Tian H, Tao Y, Reyes MP, Shyu M-L, Chen S-C, Iyengar SS (2018) A survey on deep learning: algorithms, techniques, and applications. ACM Comput Surv (CSUR) 51:1–36

[50] Chmarra MK, Grimbergen CA, Jansen F-W, Dankelman J (2010) How to objectively classify residents based on their psychomotor laparoscopic skills? Minimally Invasive Ther Allied Technol 19:2–11 10.3109/13645700903492977 20095891

[51] Chmarra MK, Klein S, de Winter JC, Jansen F-W, Dankelman J (2010) Objective classification of residents based on their psychomotor laparoscopic skills. Surg Endosc 24:1031–1039 19915915 10.1007/s00464-009-0721-y PMC2860557

[52] Rahimi AM, Hardon SF, Uluç E, Bonjer HJ, Daams F (2023) Prediction of laparoscopic skills: objective learning curve analysis. Surg Endosc 37:282–289 35927349 10.1007/s00464-022-09473-7 PMC9839814

[53] Ismail AJ, Mishra R (2014) Comparing task performance and comfort during nonpulmonary video-assisted thoracic surgery procedures between the application of the ‘baseball diamond’ and the ‘triangle target’ principles of port placement in swine models. World 7:60–65

[54] Khanfar A, Motamedi S, Safford S, Moore J, Menold J, Miller S (2025) Tracking what matters: deep learning-based AOI annotation for eye-tracking in medical training. J Surg Res (submitted)

[55] Tzamaras HM, Wu H-L, Moore JZ, Miller SR (2023) Shifting perspectives: a proposed framework for analyzing head-mounted eye-tracking data with dynamic areas of interest and dynamic scenes. Proc Hum Factors Ergon Soc Annu Meet. 10.1177/21695067231192929 38450120 10.1177/21695067231192929 PMC10914345

[56] Jongerius C, Callemein T, Goedemé T, Van Beeck K, Romijn JA, Smets E, Hillen M (2021) Eye-tracking glasses in face-to-face interactions: manual versus automated assessment of areas-of-interest. Behav Res Methods. 10.3758/s13428-021-01544-2 33742418 10.3758/s13428-021-01544-2 PMC8516759

[57] Cohen J (1960) A cofficient of agreement for nominal scales. Educ Psychol Meas 20:37–46

[58] Olsen A (2012) The Tobii I-VT fixation filter. Tobii Technology 21;4–19. https://www.tobiipro.com/learn-and-support/learn/steps-in-an-eye-tracking-study/data/how-are-fixations-defined-whenanalyzing-eye-tracking-data/

[59] Hossain A, Miléus E (2016) Eye movement event detection for wearable eye trackers. Linköpings Universitet

[60] Komogortsev OV, Jayarathna S, Koh DH, Gowda SM (2010) Qualitative and quantitative scoring and evaluation of the eye movement classification algorithms. Proceedings of the 2010 Symposium on eye-tracking research & applications, pp 65–68

[61] Specian Junior FC, Litchfield D, Sandars J, Cecilio-Fernandes D (2024) Use of eye tracking in medical education. Med Teach. 10.1080/0142159X.2024.2316863 38382474 10.1080/0142159X.2024.2316863

[62] Breiman L (2001) Random forests. Mach Learn 45:5–32

[63] Kasula BY (2019) Enhancing classification precision: exploring the power of support-vector networks in machine learning. Int Sci J Res 1

[64] Fratello M, Tagliaferri R (2018) Decision trees and random forests. Encyclopedia of Bioinformatics and Computational Biology: ABC of Bioinformatics 1

[65] Abiodun OI, Jantan A, Omolara AE, Dada KV, Mohamed NA, Arshad H (2018) State-of-the-art in artificial neural network applications: a survey. Heliyon 4 10.1016/j.heliyon.2018.e00938 PMC6260436 30519653

[66] Kokol P, Kokol M, Zagoranski S (2022) Machine learning on small size samples: a synthetic knowledge synthesis. Sci Prog 105:00368504211029777 35220816 10.1177/00368504211029777 PMC10358596

[67] Shaikhina T, Lowe D, Daga S, Briggs D, Higgins R, Khovanova N (2015) Machine learning for predictive modelling based on small data in biomedical engineering. IFAC-PapersOnLine 48:469–474

[68] Uddin S, Khan A, Hossain ME, Moni MA (2019) Comparing different supervised machine learning algorithms for disease prediction. BMC Med Inform Decis Mak 19:1–16 31864346 10.1186/s12911-019-1004-8 PMC6925840

[69] Cha G-W, Moon H-J, Kim Y-C (2021) Comparison of random forest and gradient boosting machine models for predicting demolition waste based on small datasets and categorical variables. Int J Environ Res Public Health 18:8530 34444277 10.3390/ijerph18168530 PMC8392226

[70] Wong T-T (2015) Performance evaluation of classification algorithms by k-fold and leave-one-out cross validation. Pattern Recognit 48:2839–2846

[71] Arthur D, Vassilvitskii S (2007) k-means++: The advantages of careful seeding. Soda, pp 1027–1035

[72] Cui M (2020) Introduction to the k-means clustering algorithm based on the elbow method. Accounting Auditing Financ 1:5–8

[73] Vakili M, Ghamsari M, Rezaei M (2020) Performance analysis and comparison of machine and deep learning algorithms for IoT data classification. arXiv preprint arXiv:200109636

[74] Nembrini S, König IR, Wright MN (2018) The revival of the Gini importance? Bioinformatics 34:3711–3718 29757357 10.1093/bioinformatics/bty373 PMC6198850

[75] Fernández-Delgado M, Cernadas E, Barro S, Amorim D (2014) Do we need hundreds of classifiers to solve real world classification problems? J Mach Learn Res 15:3133–3181

[76] Ziegler A, König IR (2014) Mining data with random forests: current options for real-world applications. Wiley Interdiscip Rev Data Min Knowl Discov 4:55–63

